# Misregulation of ELK1, AP1, and E12 Transcription Factor Networks Is Associated with Melanoma Progression

**DOI:** 10.3390/cancers12020458

**Published:** 2020-02-17

**Authors:** Komudi Singh, Michelle Baird, Robert Fischer, Vijender Chaitankar, Fayaz Seifuddin, Yun-Ching Chen, Ilker Tunc, Clare M. Waterman, Mehdi Pirooznia

**Affiliations:** 1 Bioinformatics and Computational Biology Laboratory, National Heart Lung and Blood Institute, National Institutes of Health, Bethesda, MD 20892, USA; komudi.singh@nih.gov (K.S.); vijender.chaitankar@nih.gov (V.C.); fayaz.seifuddin@nih.gov (F.S.); yun-ching.chen@nih.gov (Y.-C.C.); ilker.tunc@nih.gov (I.T.); 2 Cell and Developmental Biology Center, National Heart Lung and Blood Institute, National Institutes of Health, Bethesda, MD 20892, USA; michelle.baird@nih.gov (M.B.); fischerr2@nhlbi.nih.gov (R.F.); watermancm@nhlbi.nih.gov (C.M.W.)

**Keywords:** cancer progression, melanoma, co-expression network analysis, transcriptomics

## Abstract

Melanoma is among the most malignant cutaneous cancers and when metastasized results in dramatically high mortality. Despite advances in high-throughput gene expression profiling in cancer transcriptomic studies, our understanding of mechanisms driving melanoma progression is still limited. We present here an in-depth bioinformatic analysis of the melanoma RNAseq, chromatin immunoprecipitation (ChIP)seq, and single-cell (sc)RNA seq data to understand cancer progression. Specifically, we have performed a consensus network analysis of RNA-seq data from clinically re-grouped melanoma samples to identify gene co-expression networks that are conserved in early (stage 1) and late (stage 4/invasive) stage melanoma. Overlaying the fold-change information on co-expression networks revealed several coordinately up or down-regulated subnetworks that may play a critical role in melanoma progression. Furthermore, by incorporating histone lysine-27 acetylation information and highly expressed genes identified from the single-cell RNA data from melanoma patient samples, we present a comprehensive list of pathways, putative protein-protein interactions (PPIs) and transcription factor (TF) networks that are driving cancer progression. From this analysis, we have identified Elk1, AP1 and E12 TF networks that coordinately change expression in late melanoma when compared to early melanoma, implicating these TFs in melanoma progression. Additionally, the sumoylation-associated interactome is upregulated in invasive melanoma. Together, this bioinformatic analysis potentially implicates a combination of TF networks and PPIs in melanoma progression, which if confirmed in the experimental systems, could be used as targets for drug intervention in melanoma.

## 1. Introduction

Melanocytes are a population of cells that arise from the neural crest lineage and are found in the skin, the middle layer of the eye, the inner ear, the meninges, bones and the heart [1,2]. In the skin, these cells are present in the most bottom layer of the skin epidermis and in the hair follicles, and are primarily responsible for the production of the pigment melanin, which is synthesized and stored in lysosome-like organelles called melanosomes [3]. One of the main functions of the pigment melanin is to absorb ultraviolet radiation (UVR) from the sun to minimize skin damage [3]. Cutaneous melanoma is a common type of skin cancer that arises in the melanocytes, and when treated early has a high survival rate [4]. However, despite advances in our understanding of the disease progression, 20% of those diagnosed with melanoma will die from the disease [5]. The majority of cutaneous melanomas arise as a result of the proliferation of melanocytes, referred to as melanocytic naevi. These benign growths have a low likelihood of progressing to melanoma. However, increasing occurrences of these melanocytic naevi raises the chance for melanoma development [6].

A combination of genetic and environmental factors has been found to influence melanoma pathogenesis. Inactivating mutations in the cyclin-dependent kinase inhibitor 2A (CDKN2A) gene result in a dysregulated cell cycle promoting melanoma transformation [7,8]. Mutations in *NRAS*, *BRAF*, and *PTEN* genes have emerged as factors contributing to melanoma [9]. Furthermore, the presence of germline or acquired mutations in the promoter region of *TERT* gene also increases the predisposition to melanoma [10]. Environmental factors such as light skin color, lack of melanin, and excessive exposure to UVR from sun increase the risk of melanoma. Consistently, melanomas with driver gene mutations also exhibit features of DNA damage arising from UVR exposure [6]. 

While considerable data is available on the types of mutations acquired during the expansion of naevi, relatively less is known about changes in gene expression during metastasis of melanoma. Recent studies have enabled grouping melanoma samples based on the signature gene expression patterns [11] as well as construct co-expression networks to delineate hub genes and transcription factor networks likely involved in melanoma pathology [12]. However, these studies provide little information about the mechanisms and cellular processes driving tumor progression from early stage benign naevi to metastatic melanoma.

To specifically study tumor progression, we have utilized a publicly available melanoma RNA-seq data set (GEO: GSE98394) [13] comprised of data from benign, non-invasive, and invasive sample groups. Instead of comparing metastatic samples to either benign or normal samples, we use the clinical information for each of the non-invasive and invasive samples to further stratify them into 4 stages from the early non-invasive stage (stage 1) to the late metastatic stage (stage 4/invasive). We generated gene co-expression networks from the re-grouped melanoma sample RNA-seq data and used the correlated gene modules to uncover potential protein-protein interactions (PPIs) and transcription factor (TF) networks. In parallel, we have identified genes associated with super-enhancers using melanoma chromatin immunoprecipitation (ChIP-seq) data [14] and found that >20% of genes that were differentially upregulated in invasive melanoma were also associated with super-enhancers and overlapped with two PPI networks involving the sumoylation protein SUMO3 and polycomb group protein RNF2. Network analysis also revealed the potential involvement of three TFs Elk1, AP1, and E12 as the target genes in these networks that were either coordinately upregulated or downregulated in invasive melanoma. These findings suggest that a concomitant change in the transcriptional landscape resulting from mis-regulation of several TFs contributes to tumor progression in melanoma. Despite this complexity, the tumor progression mechanisms uncovered in this study could serve as nodal points of for further investigation in the biological systems and could serve as potential targets of intervention for effective cancer therapy. 

## 2. Methods

### 2.1. RNA-seq Data Grouping and Sample Selection

**Figure 1:**
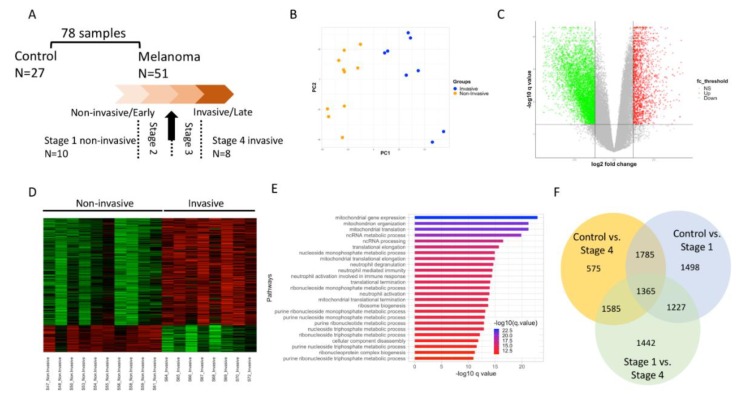
Study design and differential expression analysis between stage 1 and stage 4 to delineate melanoma cancer progression. (**A**). 51 melanoma samples were further stratified to identify sample groups that represents stage 1 and stage 4 melanoma. (**B**). Principal component analysis (PCA) of DE genes identified in stage 1 vs stage 4 DE analysis. (**C**). Volcano plot revealing upregulated (red color dots) and downregulated (green color dots) genes from stage 1 vs stage 4 comparison. (**D**). Heatmap of top 200 DE genes from stage 1 vs stage 4 DE analysis. (**E**). Pathway enrichment analysis of DE genes from stage 1 vs stage 4 DE analysis. Overlap of DE genes from three-way comparison of indicated groups. DE: differentially expressed. (**F**). Overlap of DE genes from three-way comparison of indicated groups. DE: differentially expressed.

Seventy eight (78) RNA-seq data downloaded from GEO: GSE98394 [13] have been categorized into benign (27 samples) and two stages of melanocytic tumors: non-invasive (35 samples), and invasive (16 samples). To further delineate non-invasive and invasive tumor stages, the tumor groups were further categorized into smaller groups that would clinically represent non-invasive (stage 1) and invasive (stage 4) tumor stages (Figure 1A). The clinical phenotype and staging information are summarized in (Table S1). Specifically, the non-invasive melanoma group represented tumors with thickness less than 1mm and were categorized into the T1a and stage I based on TNM Classification of Malignant Tumors (TMN) [15] and American Joint Committee on Cancer (AJCC) classification [16], respectively. The invasive melanoma group on the other hand represented tumors with thickness > 4 mm. The tumors were classified as T3/T4 and stage III based on TMN and AJCC classification, respectively. The majority of the subjects in this group were deceased. Additionally, this sub-group of invasive cancer subjects had at least one lymph node that contained cancer while subjects with non-invasive tumors had no cancer in the lymph nodes, and in one case the cancer in the lymph node could not be determined (Table S1). The sample size of the non-invasive (stage 1) and invasive (stage 4) cancer samples that were re-categorized based on the phenotypic information was 10 and 8 samples, respectively.

### 2.2. RNA-seq Analysis 

The RNA sequence data was subjected to quality control using FastQC (v0.11.8) [17], followed by alignment to the human genome (GRCh38) using spliced transcripts alignment to a reference (STAR) [18]. Gene expression was quantified using featureCounts (v2.0.0) [19]. The tumor purity of the RNAseq samples were assessed using ESTIMATE (v 1.0.13) [20] R package and the tumor purity estimate, and sex was used as covariates and differential expression analysis was performed using limma-voom [21]. The tumor purity estimates for stage 1 and stage 4 samples cis presented in Table S2. The statistically significant differentially expressed genes with *p* value < 0.05 were further subjected to pathway enrichment analysis using clusterProfiler (v3.14.3) [22]. A summary of the analysis pipeline is presented as a flow-chart in Figure S1.

### 2.3. ChIP-seq and Super-Enhancer Analysis 

The superenhancer analysis was performed on melanoma samples of the CHL-1 cell lines originating from skin tissue of human samples. To select the samples, we queried Cistrome database [23] for samples with keyword “melanoma” and filtered the results for human samples specific to H3K27ac assays. Cistrome is the largest known database that holds uniformly processed data for ChIP data sets. A total of 10 samples was returned by Cistrome database, of which three passed rigorous QC analysis of six different parameters which indicates that we were very stringent in our sample selection process. These three samples were also published in [24].

Raw FASTQ files (GEO: GSE60666) [14] were downloaded from GEO [25], and each file was initially trimmed for adapters, and low-quality reads using Trimmomatic (v0.39) [26] with the following parameters ‘slidingwindow: 4:15 minlen: 36’. Trimmed reads were then aligned to the human reference genome *hg38* using the BWA-backtrack utility (v0.7.17) [27]. High-quality alignments were retained with the SAMtools program [28] using the following parameters ‘-q 30 -F 772’. Duplicate alignments were purged using SAMtools ‘rmdup’ utility. High quality and unique alignments in ENCODE’s blacklisted regions were purged using the BEDTools program (v2.29.0) [29]. Seven of 11 samples produced ≥20 million high-quality alignments. Enriched regions for the histone lysine 27 residue acetylation H3K27ac mark in these seven samples were identified using the MACS2 peak finding tool (v2.2.5) [28]. Four of these samples showed high concordance based on Jaccard’s similarity metric [30]. Alignments of these four samples were merged using SAMtools merge utility (v1.9), and homer tool [31] was used to identify super-enhancers. We identified super-enhancer associated genes by overlapping the transcription start sites of genes that fall within ±10 kb of the super-enhancers.

### 2.4. Co-Expression Network Analysis

The RNA-seq data was filtered to select genes that had expression gene count > 5 in 90% of the samples to remove low-expressing genes. The filtered RNA-seq data was subjected to weighted gene co-expression network analysis (WGCNA) [32]. A consensus WGCNA of the non-invasive (stage 1) and invasive (stage 4) RNA-seq data combined together helped identify 50 co-expression modules with a Pearsons’ correlation cutoff of 0.8. Of these modules, the modules with > 150 genes overlap with DE genes were used for subnetwork analysis. These modules were subjected to GeneMANIA [33] database search in cytoscape to identify potential protein-protein interactions (PPIs) and transcription factors (TFs). The resulting PPI and TF target networks were overlaid with the gene expression fold change information using Cytoscape [34].

### 2.5. Single-Cell RNA-seq (scRNA-seq) Analysis

The expression matrix was obtained from the Tirosh, et al. melanoma single-cell RNA-seq study [35]. We compiled a list of 547 genes highly expressed in malignant melanoma cells in each sample compared with non-malignant cells (FDR < 0.05).

## 3. Results

### 3.1. Sample Processing and Differential Expression Analysis to Understand Melanoma Cancer Progression

Gene expression was computed for a total of 78 RNA-seq samples (27 benign melanocytes, 35 non-invasive melanomas, and 16 invasive melanomas). Principal component analysis (PCA) on gene expression profiles barely separated non-invasive from invasive melanomas (Figure S2). This suggested that melanomas remained heterogeneous with the current grouping and may require a refined grouping to obtain better contrast in gene expression associated with invasiveness. Additionally, ambiguity in assigning stage information to close categories of samples could also add to the discrepancy. To this end, we reclassified melanoma samples into 4 stages using clinical phenotypes pertinent to melanoma invasiveness (see Methods), with the early non-invasive samples at stage 1 (*n* = 10) and the late invasive samples at stage 4 (*n* = 8) and these were processed for downstream analysis (Figure 1A). The clinical phenotype and staging information are summarized in (Table S1 and methods). The PCA of the regrouped samples showed clear separation between samples in stage 1 and stage 4 indicating that transcriptomic differences in these samples are likely driven by tumor stage (Figure 1B). Differential expression (DE) analysis identified statistically significant changes in gene expression between stage 1 and stage 4 samples and the fold change of upregulated and downregulated genes are depicted in the volcano plot (Figure 1C). A heat map of the top 200 DE genes subset based on *q* value < 0.05 is shown in Figure 1D. The DE genes were significantly enriched for several mitochondrial genes, for mitochondrial translation, and RNA metabolism-related processes (Figure 1E).

In addition to the early (stage 1) vs. late-stage (stage 4) melanoma analysis, differential gene expression analysis was also performed for two other comparisons including the benign (27 samples) vs. non-invasive tumor (10 samples) [Control vs. Stage 1] and benign vs. invasive tumor (8 samples) [Control vs. Stage 4]. The PCA, volcano plot and heatmap revealed distinct gene expression patterns between the different groups under comparison (Figures S3A–D and S4A–D).

The DE analysis revealed a large number of genes in all three comparisons, which is consistent with previously reported cancer-associated genes (see Figure 1F and [13]). However, of the differentially expressed genes, only a small proportion were common between the above-described comparisons (Figure 1F). These data suggest that distinct gene expression changes occur in stage 1 and stage 4 melanoma, which is likely representative of the respective tumor stage.

### 3.2. Potential Link between Upregulated DE Genes and Super-Enhancers

**Figure 2:**
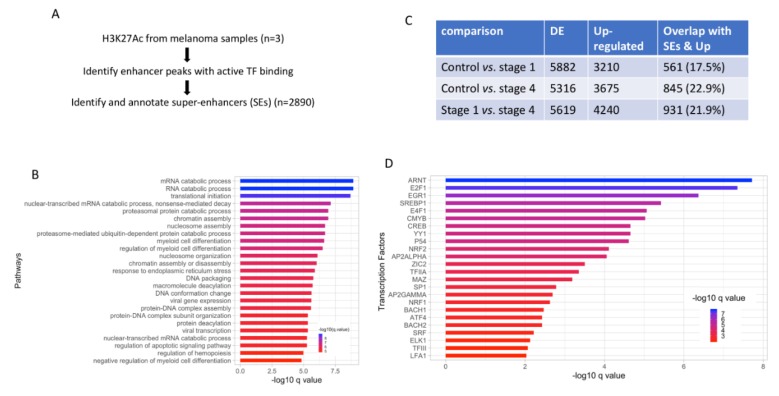
Summary of differential gene expression and super-enhancer analysis. (**A**). A flow diagram summarizing the ChIP-seq data analysis from melanoma samples. (**B**). Pathway enrichment analysis of SE associated genes. (**C**). Table showing overlap between SE associated genes and upregulated DE genes from the indicated comparisons. (**D**). TF enrichment analysis of SE associated genes to identify potential TFs regulating these genes. DE: differentially expressed; TF: Transcription factor; SE: super-enhancer.

Similar to gene expression changes, changes to the epigenome are widely accepted to be a critical process driving cancer cell transformation [36,37]. To further understand the role of DE genes in melanoma pathology, their potential link to histone modifications, specifically the histone acetylation at lysine 27 residue was explored. Since histone acetylation at lysine 27 residue is associated with transcriptionally active DNA regions, their presence could be assessed to identify segments of DNA regions that are robustly bound by transcription factors known as super-enhancers (SE). ChIP-seq analysis of melanoma samples helped identify 2890 genes with putative association with super-enhancers (Figure 2A). First, the pathway enrichment analysis of genes associated with super-enhancers showed significant enrichment of genes in the processes associated with mRNA processing (Figure 2B).

Next, the ratio of DE genes that both overlapped with SE associated genes and were also upregulated in each of the three comparisons were analyzed. In all three comparisons, 18–20% of upregulated DE genes from invasive versus control or non-invasive samples overlapped with super-enhancers, suggesting that epigenetic changes to some extent may underlie misregulation of gene expression in melanoma (Figure 2C). Furthermore, transcription factor (TF) enrichment analysis of SE associated genes showed that a wide group of TFs target the SE associated genes (Figure 2D). Of these, TFs AP2 alpha, AP2 gamma are known to be upregulated in carcinoma and E2F1 is a known regulator of keratinocyte proliferation [38]. One limitation of this analysis is that the scope of the transcriptional landscape change in melanoma cannot be fully evaluated as the samples used in the ChIP were not stratified in the same way as the RNAseq data (see Discussion).

### 3.3. Co-Expression Network Analysis to Understand Cancer Progression

**Figure 3:**
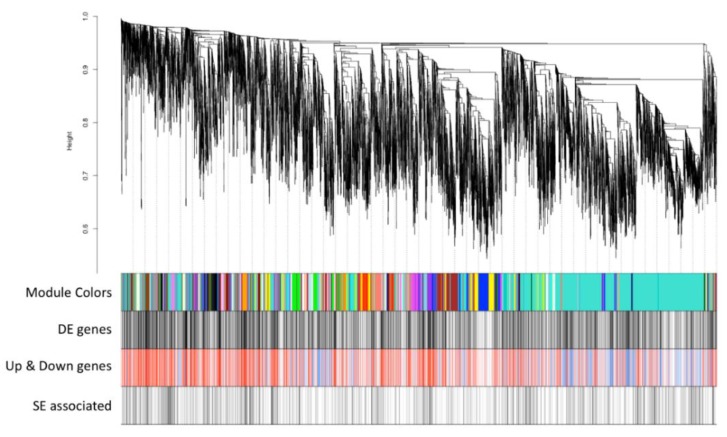
Overlap of consensus co-expression modules with DE genes and super-enhancer associated genes. Cluster dendrogram of co-expressing genes from consensus WGCNA analysis (see methods). Each color represents genes with correlated gene expression in invasive (stage 4) and non-invasive (stage 1) RNA-seq data. The DE genes are marked in a block as black color vertical lines (underneath the cluster dendrogram). Of these genes that are upregulated and downregulated are colored as red and blue, respectively, and this panel is labeled as “Up & Down genes”. The last block represents SE associated genes. DE: differentially expressed; SE: super-enhancer.

To further understand the potential mechanisms underlying melanoma cancer pathogenesis and progression, the invasive (stage 4/late) and the non-invasive (stage 1/early) melanoma RNA-seq data were subjected to the consensus weighted gene co-expression network analysis (WGCNA). This analysis resulted in the identification of 50 modules with genes that have correlated expression in both early (stage 1) and late (stage 4) melanoma, which are shown as a cluster dendrogram where the cluster/modules of correlated genes across 18 samples are color-coded (Figure 3 and Figure S5). The number of DE genes and the number of genes associated with super-enhancers in each cluster is summarized in Table S8. This analysis revealed that the turquoise module has the biggest overlap with DE genes as well as with the super-enhancers. Furthermore, five additional modules—black, blue, brown, red, and yellow modules were identified to overlap with more than 150 DE genes each. These modules were selected and subjected to sub-network analysis (described below). Finally, to assess the extent to which the consensus gene co-expression modules overlapped with either the non-invasive or invasive RNA-seq data co-expression modules, the modules across the data sets were cross-compared. First, the consensus turquoise module showed significant overlaps with three invasive melanoma gene co-expression modules. We chose an arbitrary overlap that was greater than 200 genes in size and assessed them by pathway enrichment (Figure S5A). Analysis of overlaps smaller than 200 genes is not reported as they did not produce statistically significant pathway enrichment results in most cases. Grey modules from either dataset were excluded from this evaluation as this module represents uncorrelated genes. Overlapping genes were broadly enriched for processes related to histone modification, mRNA regulation, vacuole and autophagy (Tables S3.1–S3.3). Similarly, gene overlaps between consensus modules and non-invasive RNA-seq data were analyzed (Figure S5B). One consensus module (turquoise) exhibited overlap with six non-invasive co-expressing modules. Common genes between the indicated modules were further assayed for pathway enrichment and exhibited either no significant enrichment (one consensus module and non-invasive green modules) or enrichment in processes related to histone and mRNA regulation, which was similar to that found with the invasive RNA-seq data (Tables S3.4–S3.8). This analysis showed that all of the consensus co-expressing modules (except turquoise modules) overlapped to a small extent with co-expressing modules from either invasive or non-invasive RNA-seq data. These observations suggest that genes in the consensus modules represent genes clusters conserved in early (stage 1) and late (stage 4/invasive) melanoma stages and are likely to be associated with melanoma progression rather than representing either invasive or non-invasive stages of melanoma. It is likely that the consensus modules will provide insight into the processes underlying cancer progression.

To test this, the genes in the consensus modules were subjected to sub-network analysis. Consensus WGCNA analysis of invasive and non-invasive RNA-seq data helped identify 50 modules, each comprising of genes that have correlated expression pattern (See Figure 3). Table S8 shows the extent of overlap of modules with DE genes. About 25% of the statistically significant DE genes were assigned to the uncorrelated “grey” module and were excluded from pathway enrichment and subnetwork analysis. Since these six modules with DE gene overlap >150 accounted for ~65% of the DE genes assigned to all the correlated modules, they we selected for downstream analysis. The DE genes from these selected modules were subjected to pathway enrichment analysis and were further analyzed by GeneMANIA to identify potential protein-protein interactions (PPIs) and potential transcription factors (TFs) that may be regulating the co-expressing genes. The pathway enrichment analysis of the genes in the two (red and black) modules showed enrichment in processes associated with the extracellular matrix and no significant enrichment, respectively. A summary of the findings for the remaining four modules is presented below.

### 3.4. Sumoylation and ELK1 TF Targets Interactome is Highly Expressed in Invasive Melanoma

**Figure 4:**
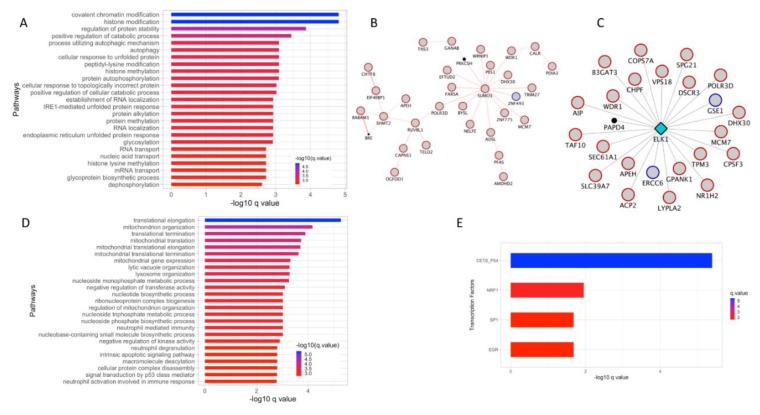
WGCNA analysis of stage 1 vs. stage 4 melanoma, turquoise module. (**A**). Pathway enrichment analysis of DE genes that overlapped with the turquoise module. (**B**). PPI analysis using GeneMANIA revealed an interactome associated with sumoylation processes. Red color boundary represents genes upregulated in invasive melanoma compared to non-invasive melanoma. (**C**). TF network analysis using GeneMANIA revealed a TF network driven by Elk1 transcription factor. Red color boundary represents genes upregulated in invasive melanoma compared to non-invasive melanoma. (**D**). Pathway enrichment analysis of SE associated genes that overlapped with turquoise module. (**E**). TF enrichment analysis using GSEA C3 motif gene set of super-enhancers associated turquoise module genes. (**F**). TF networks identified in (**E**) is represented as a network where the diamond-shaped hub in the TF and the spokes connect with the targets. Red and blue color boundary represents genes upregulated and downregulated, respectively, in invasive melanoma compared to non-invasive melanoma. DE: differentially expressed; SE: super-enhancer.

The genes in the turquoise module showed the biggest overlap with DE genes from invasive vs. non-invasive RNA-seq data comparison. First, pathway enrichment analysis of the DE genes from this module showed significant enrichment in processes associated with histone modification, protein modification, and unfolded protein response (Figure 4A and Table S4.1).

Next, analysis of these genes in GeneMANIA for PPIs helped uncover an interactome with the SUMO3 as the hub gene. SUMO3 is a small ubiquitin-like protein that can modify other proteins by covalently binding to the lysine residues of the target protein. Finally, the gene-expression fold change information was overlaid on top of this interactome. The resulting network with gene expression fold-change information indicated that SUMO3 gene and most of its interactors are up-regulated in invasive melanoma when compared with non-invasive melanoma (Figure 4B). Additionally, GeneMANIA analysis helped identify ELK1 as a potential TF regulating several DE genes from this module (Figure 4C). When the gene expression fold change information was superimposed on to the *ELK1* target genes, the resulting network indicated that a majority of the ELK1 targets are upregulated in invasive melanoma.

### 3.5. Super-Enhancer (SE) Associated Genes from the Turquoise Module Implicate Four TFs in Melanoma Progression

The genes in the turquoise module also showed the biggest overlap with genes associated with SE. Pathway enrichment analysis was performed on common genes from this module that also overlapped with genes associated with SE. Some of the significant pathways included lysosome, vacuole, pigment granules and melanoma organization (Figure 4D). Since the super-enhancers are transcriptionally active DNA regions most likely associated with TFs, these genes were subjected to TF enrichment analysis using the transcription factor target gene set downloaded from the molecular signature database (MSigDB). This analysis identified 4 TFs whose targets overlapped with genes present in this module (Figure 4E and Table S4.2). When the gene expression fold change information was superimposed on top of these TF networks, a majority of the target genes are upregulated in invasive melanoma when compared to the non-invasive melanoma. The TFs in the networks are represented as the diamond shaped hub and the nodes connected by spokes to the hub represent target genes (Figure S6A–D). In all four TF networks, the target genes were mostly upregulated in invasive melanoma (Figure S6A–D).

### 3.6. Protein Ubiquitination Process and ELK1 TF Targets Are Upregulated in Invasive Melanoma

**Figure 5:**
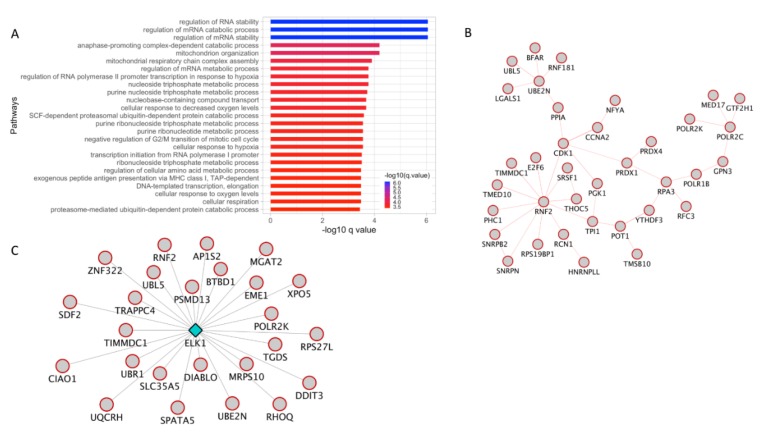
WGCNA analysis of stage 1 vs**.** stage 4 melanoma, blue module. (**A**). Pathway enrichment analysis of DE genes that overlapped with the genes in the blue module. (**B**). PPI analysis using GeneMANIA revealed an interactome with RNF2 protein as hub. Red color boundary represents genes upregulated in invasive melanoma compared to non-invasive melanoma. (**C**).TF network analysis using GeneMANIA revealed a TF network driven by Elk1 transcription factor. Red color boundary represents genes upregulated in invasive melanoma compared to non-invasive melanoma. DE: differentially expressed; TF: transcription factor.

The genes in the blue module were significantly enriched in the pathways associated with mRNA stability, cell cycle, and protein ubiquitination processes (Figure 5A and Table S5). GeneMANIA analysis of the genes in this module lead to identification of PPI network with RNF2 gene as the hub (Figure 5B). RNF2 encodes a ubiquitin ligase and plays a central role in gene expression regulation [39,40]. In addition to the PPI, GeneMANIA analysis of the genes in the blue module also identified ELK1 TF as a potential transcriptional regulator for the genes in the blue module. When gene expression fold-change information was overlaid on top of the ELK1 TF network, it indicated that all of the target genes are upregulated in invasive melanoma (Figure 5C). Consistent with this observation, ELK1 TF network identified in the turquoise module were also mostly upregulated in invasive melanoma (Figure 4C).

### 3.7. Co-Expression Module Associated with Skin/Keratinocyte Associated Processes

**Figure 6:**
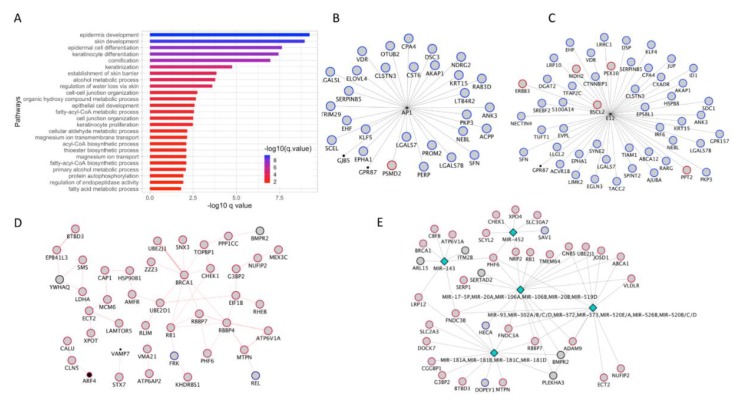
WGCNA analysis of stage 1 vs. stage 4 melanoma, yellow and brown module. (**A**). Pathway enrichment analysis of DE genes that overlapped with the yellow module. (**B**,**C**). TF network analysis using GeneMANIA revealed two TF networks each driven by AP1 and E12 transcription factors. (**D**,**E**). PPI (involving BRCA1) and miRNA networks identified by GeneMANIA analysis of genes in the brown nodule. The miRNAs are represented as diamond shaped nodes. Red and blue color boundary represents genes upregulated and downregulated, respectively, in invasive melanoma compared to non-invasive melanoma. DE: differentially expressed; TF: transcription factor. PPI: protein-protein interaction.

Analysis of the genes in the yellow module helped uncover a module comprised of genes involved in skin, epidermis, and keratinocyte development (Figure 6A, Table S6). This was evident as the genes in this module were enriched for processes associated with epidermis development and differentiation, which is the layer of skin that harbors melanocytes.

GeneMANIA analysis of the genes in this module identified two TFs AP1 and E12 that regulated a broad number of genes in this module (Figure 6B,C). When the gene expression fold-change information was overlaid on these TF networks, it indicated that the target genes are mostly downregulated in both of these TF networks.

### 3.8. Identification of BRCA1 Hub and Involvement of MicroRNA

In the Brown modules, the PPI analysis revealed a BRCA1 interaction hub, where BRCA1 and its interacting partners were upregulated in late (stage 4) melanoma versus early (stage 1) melanoma. BRCA1 protein with mutations is known to have a shorter half-life, which results in impaired DNA damage repair and tumor progression [41,42]. Given this function of *BRCA1*, it is likely that the upregulation of *BRCA1* gene expression could be a compensatory mechanism in response to melanoma progression. In addition to *BRCA1* protein-protein interactome, several microRNA targets were also identified in this module. The coordinated up-regulation of a majority of these microRNA targets suggest a potential role for microRNAs in melanoma biology.

### 3.9. Single Cell (sc) RNA-seq Analysis of Melanoma Overlaps with Genes Upregulated with Stage 4 Melanoma

**Figure 7:**
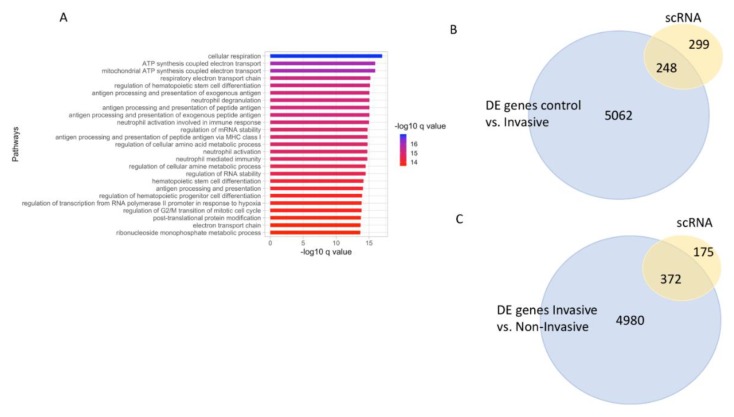
Overlap of highly expressed genes identified from melanoma single cell (sc) RNA-seq data. (**A**). Pathway enrichment analysis of highly expressed genes identified from scRNA-seq melanoma data. (**B**). Overlap of highly expressed genes identified from melanoma scRNA data with DE genes from invasive vs. non-invasive comparisons. (**C**). Overlap of highly expressed genes identified from melanoma scRNA data with DE genes from control vs. invasive comparisons.

To further assess the gene expression patterns in the melanoma samples, scRNA-seq data from melanoma samples were analyzed (see methods). This analysis revealed a group of genes that were highly expressed in the melanoma samples (Table S7). Pathway enrichment analysis of these genes revealed enrichment of pathways involved in cellular respiration, mRNA processing, and several processes associated with oxygen sensing (Figure 7A and Table S7). Furthermore, a majority (69%) of these highly expressed genes from scRNA-seq data also overlapped with DE genes identified in stage 1 vs. stage 4 melanoma comparison (Figure 7B). On the other hand, 48% of the highly expressed genes overlapped with DE genes identified in control (benign) vs. late stage melanoma (Figure 7C). Additionally, all of the 378 genes from scRNA-seq analysis that overlapped with the DE genes from invasive vs. non-invasive comparison (Figure 7B), also has their expression count level > 50 counts, and 373 overlapping genes were upregulated in invasive (stage 4) melanoma. Combined, this analysis suggests that the highly expressed genes identified from the sc RNA-seq data is representative of highly differentially upregulated genes in stage 4 melanoma.

## 4. Discussion

Melanoma, when metastasized, is one of the deadliest forms of skin cancer, with high rates of mortality. Several studies have dissected the genomic mutation load and their association with melanoma progression. Additionally, a handful of studies have also looked at the gene expression profile by comparing the benign melanoma samples with that of metastatic cancer. While these studies have provided insights into a large number of differentially expressed (DE) genes and the pathways in which these genes enrich, little can be inferred about the mechanisms underlying melanoma tumor progression. To delineate processes associated with tumor progression, using the RNA-seq data, we have performed consensus network analysis that involves building co-expression gene networks conserved in both non-invasive and invasive stages of melanoma. Unlike past studies, we have incorporated all the relevant clinical data associated with the available melanoma samples to re-group the samples into stage 1 and stage 4 tumor, to precisely capture gene expression changes associated with melanoma cancer progression. Furthermore, we have incorporated the super-enhancer (SE) associated genes from the ChIP seq data to the co-expression networks to get a comprehensive understanding of how coordinate changes in gene expression contribute to tumor progression. This analysis revealed a number of TF and PPI networks that may facilitate melanoma cancer progression. Further experimental evidence will, however, be required to test if these networks are critical for melanoma. Nevertheless, our approach of incorporating clinical information for sample stratification and analysis could be effectively applied to other cancer types to get new insights in cancer biology.

To specifically address melanoma tumor progression, we have incorporated relevant clinical information, to further sub-group the melanoma cancer samples into stage 1 (early) and stage 4 (late/invasive) melanoma representing advanced stage metastasized cancer, respectively. This comparison is different from the benign to malignant melanoma comparisons that have been published elsewhere [12] and allowed us to specifically compare gene expression changes that have occurred as the tumor progresses from stage 1 (early) to stage 4 (late/invasive) of malignancy. Additionally, to further our understanding of the gene expression changes, chromatin immunoprecipitation (ChIP) data from melanoma samples were analyzed to identify genes associated with SEs, which are most likely actively transcribed and over-expressed in the cells. About a third (33.2%) of the SE associated genes were also upregulated in stage 4 melanoma. One of the limitations of this analysis is that it does not reveal the full extent of the overlap between SE genes and DE genes as the samples for the ChIP seq data were not stratified as the samples of the RNAseq data (see methods). Future studies which incorporates patient clinical data to stage the samples and perform ChIP seq analysis will be needed to fully understand the epigenetic changes occurring as melanoma progresses. Nonetheless, our analysis of the melanoma ChIP seq data suggests that chromatin homeostasis is disrupted in invasive melanoma, which is consistent with our understanding that all cancers have an epigenome that is distinct from normal cells [43].

For an in-depth analysis of the gene expression data from early (stage1) and late (stage4/invasive) melanoma, the RNA-seq data of the re-categorized melanoma samples (discussed above) were subjected to consensus weighted gene co-expression network analysis (WGCNA), which allowed us to identify clusters of genes with correlated expression pattern that are also conserved in early and late melanoma gene expression datasets. We should note that these clusters of correlated genes are more likely to play a coordinated role in same/similar biological processes in the cells. However, they reveal little information about whether these genes can be implicated in melanoma progression. We address potential link between the correlated gene clusters and melanoma progression by identifying subnetworks of DE genes and by overlaying the fold change information on these subnetworks to infer up or down regulation. Fifty co-expression modules were identified, of which the subnetwork analysis of six modules were reported as they represented a majority of the DE genes identified in the RNA-seq analysis. First, pathway enrichment analysis of the co-expression modules revealed that genes in distinct modules enriched in different aspects of cellular functions. Genes in the turquoise module enriched in the processes associated with histone modification and gene expression. Consistent with the pathway enrichment, a majority of the DE genes in this module also overlapped with genes associated with SE and were upregulated in stage 4 melanoma. Additionally, genes in the blue module enriched in processes associated with protein stability and ubiquitination process. The genes in the yellow module enriched in processes associated with skin development and keratinocyte proliferation and differentiation.

Next, to further our understanding of the co-expression clusters, the DE genes in each of these clusters were analyzed by GeneMANIA to identify protein-protein interactions (PPIs) and transcription factors (TFs) that potentially regulate these correlated genes. The genes in the turquoise module were found to be associated with four TF networks, which is consistent with their potential association with SE (see results). When these TF networks were superimposed with the expression fold-change, it revealed that all four of the TF networks were upregulated in stage 4 melanoma. Of these, EGR and SP1 TFs have been shown to increase inflammation [44,45]. Furthermore, ETS1 belongs to the ETS family of TFs and has been implicated in tumorigenesis [46]. Additionally, many of the upregulated DE genes in the turquoise and blue module were also identified as targets of ELK1 TF. Interestingly, ETS1 and ELK1 have been shown to coordinately upregulate *CIP2A*, which encodes cancerous inhibitor of protein phosphatase 2A and leads to progression of gastric, cervical and breast cancer [47]. Our analysis implicates *ETS1* and *ELK1* in melanoma progression. However, further testing of these findings in the biological systems will be required to confirm their involvement in this process. Additionally, the E12 TF identified in the yellow module is a negative regulator of cell proliferation [48,49]. The E12 TF target genes were mostly downregulated in stage 4 melanoma suggesting that a loss of E12 activity and the associated loss of proliferation-limiting function may be critical for melanoma progression. The AP1 TF binding motifs have been shown to overlap with the microphthalmia-associated transcription factor (MITF), which is a master regulator of melanocytes, and AP1 has been shown to play a critical role in cancer invasion [14,50]. This and other studies support a more complicated role for the AP1 TF due to its involvement in both cell apoptosis and proliferation [51,52]. Specifically, the c-Jun AP-1 family member has been implicated in tumor progression [53], while the JUNB and JUND played tumor suppressor role [54,55]. Consistently, JUND was shown to function as a negative regulator of Ras mediated cell proliferation [56]. Notably, both *JUNB* and *JUND* are downregulated in invasive/late (stage 4) melanoma compared to stage 1 melanoma, and several DE genes from stage 1 vs. stage 4 comparison enriched in MAP kinase and Ras GTP binding processes (Table S4.1). It is likely that the loss of the apoptotic/tumor suppressor functions of AP1 may be critical for melanoma progression. These findings suggest that massive changes in the transcriptional landscape and an induction in TF activity is associated with melanoma cancer progression. Concurrent with this idea, another study implicated a bigger role for the changes in the transcriptional landscape over the presence of genetic mutations in determining the invasive state of cells in melanoma [14]. Since the TF networks were identified from the consensus co-expression networks analysis of stage 1 and stage 4 melanoma data, which showed minimal overlap with either dataset alone (See Figure S4), it is likely that these TF networks misregulation identified in this analysis, are the contributing factors of melanoma progression. However, further experimental evidence will be required to confirm that these changes are causal factors and not an end result of melanoma progression.

In addition to the TF networks, we have also identified several PPIs from various co-expression modules. Of note is the PPI identified in the turquoise module where the sumoylation protein SUMO3 forms the hub. SUMO proteins are known to modify a large number of proteins [57]. Several components of the sumoylation are upregulated in cancer [58,59]. Identification of a SUMO protein associated PPI suggests that this process may also be critical in melanoma progression. However, the involvement of the Sumoylation process in melanoma progression may be complex. This is because Sumoylation defective MITF has been shown to increase renal and melanoma carcinoma [60]. Another PPI identified in the blue module has an E3 ubiquitin ligase encoding RNF2 and Ubiquitin conjugating enzyme, UBE2N as hub proteins. Ubiquitin and ubiquitin like proteins has been shown to play a critical role in DNA repair. Misregulation of these genes may result in genomic instability [61]. Consistently, previous publications have delineated the role of RNF2 in esophageal carcinoma growth [62]. Taken together, the PPI network uncovered in this module may suggest additional roles for RNF2 in melanoma cancer. We find that the genes in this interactome is mostly upregulated in stage 4 melanoma. Whether upregulation of these genes in melanoma directly cause genomic instability and facilitate tumor progression will require further experimental analysis. Together, these findings show that ubiquitination and sumoylation processes may be critical in melanoma biology.

To further our understanding of gene expression changes in melanoma, we also used the single cell (sc) RNA-seq data from melanoma to identify a group of highly expressed genes. Interestingly, pathway enrichment analysis of these genes showed significant enrichment in processes associated with cellular respiration and oxygen-sensing. This is consistent with the potential involvement of reactive oxygen species (ROS) and mitochondrial respiration in melanoma [63,64]. We also found a majority of the highly expressed genes from scRNA data to overlap with the DE genes that were upregulated in stage 4 (late/invasive) compared to stage 1 melanoma. Since the highly expressed genes intersected with the DE genes upregulated in stage 4 (late/invasive) melanoma, little can be inferred about their potential involvement in cancer progression. However, this data supports a potential role for mitochondria and respiration in melanoma pathogenesis. Future scRNA seq experiments on staged melanoma samples will help test if the genes associated with the mitochondrial biology are necessary in all stages of melanoma or if these genes expression become prominent after melanoma progression.

## 5. Conclusions

In summary, we present here a comprehensive bioinformatic analysis of the melanoma RNA-seq data that incorporates (a) clinical information to stratify the samples into stages of melanoma cancer (b) subnetwork analysis on clusters of correlated genes (c) overlays genes associated with superenhancers from ChIP seq data. Reclassifying the melanoma samples based on the clinical information resulted in 10 stage 1 (early) and 8 stage 4 (late/invasive) samples. While these samples (total = 18 samples) were sufficient to create robust correlated clusters, future studies on larger cohort of staged melanoma samples will be needed to test if similar TF and PPI subnetworks are identified to further substantiate the findings from this study. Additionally, to make such cross-sectional study effective, ChIP seq and scRNA seq analysis on clinically staged melanoma sample will be needed. As sequencing technology gets advanced and cheaper, we believe that such experiments and data will become more widely available in the future. Despite these limitations, this study provides extensive information on potential protein-protein interactions (PPIs) and transcription factor (TF) networks that might be in critical in melanoma progression. These findings suggest that melanoma progression is a complex process orchestrated by concomitant changes in TF activities and PPI networks. The findings from this study if confirmed in experimental systems will not only enhance our understanding of melanoma progression but also provide potential targets to design drug interventions. We also believe that our approach of incorporating relevant clinical information for sample classification and the downstream network analysis could be applied to other cancer data to gain more insights into cancer biology from a data science perspective and also to generate hypothesis that can be tested by biologists in experimental systems.

## Supplementary Materials

The following are available online at https://www.mdpi.com/2072-6694/12/2/458/s1, Figure S1: Flow chart of the analysis pipeline, Figure S2: PCA plot of DE gene’s expression data from unfiltered invasive versus non-invasive RNA-seq analysis, Figure S3: RNA-seq analysis of control versus stage 1 (non-invasive/early), Figure S4: RNA-seq analysis of control versus stage 4 (invasive/late), Figure S5: Overlap of consensus co-expression modules with invasive and non-invasive RNA-seq modules, Figure S6: TF networks identified from genes in the Turquoise module that overlapped with SE genes, Table S1: Sample IDs, clinical data, and their classification, Table S2.1: Pathway enrichment result for common genes between consensus turquoise and invasive brown module, Table S2.2: Pathway enrichment result for common genes between consensus turquoise and invasive turquoise module, Table S2.3: Pathway enrichment result for common genes between consensus turquoise and invasive blue module, Table S2.4: Pathway enrichment result for common genes between consensus turquoise and non-invasive turquoise module, Table S2.5: Pathway enrichment result for common genes between consensus turquoise and non-invasive blue module, Pathway enrichment result for common genes between consensus turquoise and non-invasive red module, Table S2.6: Pathway enrichment result for common genes between consensus turquoise and non-invasive red module, Table S2.7: Pathway enrichment result for common genes between consensus turquoise and non-invasive yellow module, Table S2.8: Pathway enrichment result for common genes between consensus turquoise and non-invasive brown module, Table S3: Pathway enrichment result for DE genes in the turquoise module, Table S4: Transcription factor enrichment result for genes in turquoise module that overlapped with SE associated genes, Table S5: Pathway enrichment result for DE genes in the blue module, Table S6: Pathway enrichment result for DE genes in the yellow module, Table S7: Pathway enrichment analysis of the highly expressed genes from scRNA seq data, Table S8: Overlap of genes from indicated modules with DE genes and SE genes.

## Abbreviations

AJCC	American Joint Committee on Cancer
ChIP	chromatin immunoprecipitation
DE	differential expression
GEO	gene expression omnibus
MsigDB	molecular signature database
PPI	protein-protein interaction
RNA	ribonucleic acid
sc	single cell
Seq	sequence
SE	super-enhancer
TF	Transcription factor
WGCNA	weighted gene co-expression network analysis
