# Supplementary material for: Misregulation of ELK1, AP1, and E12 Transcription Factor Networks Is Associated with Melanoma Progression
Source: Cancers (Basel). 2020 Feb 17;12(2):458. doi: 10.3390/cancers12020458 (PMC7072154; doi:10.3390/cancers12020458)
Supplement: Supplementary file 1 [file cancers-12-00458-s001.zip › cancers-701483-suppl-final/cancers-701483-suppl.docx]

Supplementray Matirials: Mis-regulation of ELK1, AP1, and E12 transcription factor networks is associated with melanoma progression

Komudi Singh, Michelle Baird, Robert Fischer, Vijender Chaitankar, Fayaz Seifuddin, Yun-Ching Chen, Ilker Tunc, Clare M. Waterman and Mehdi Pirooznia


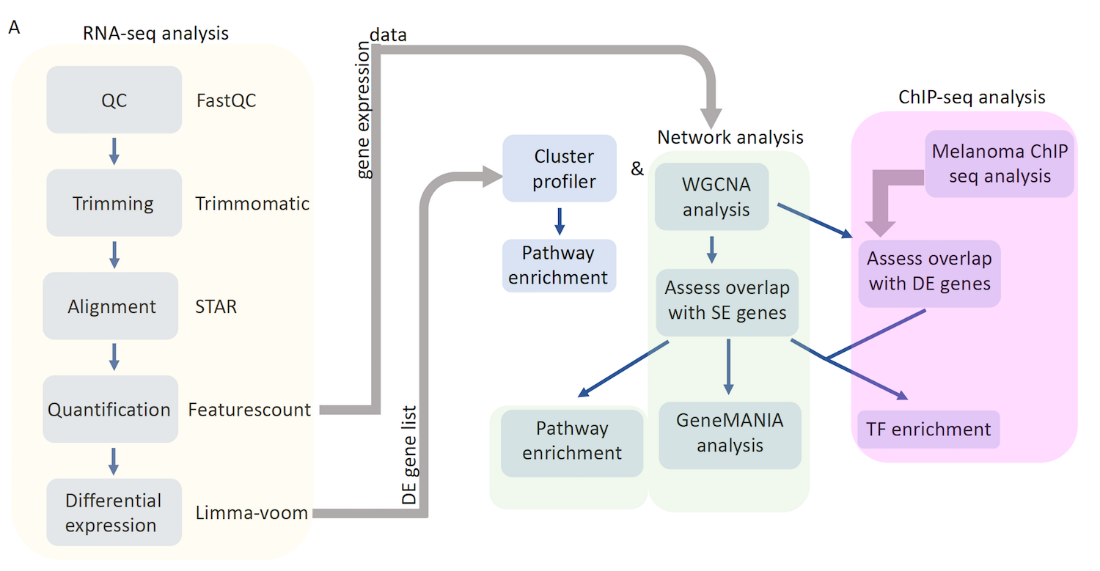


**Figure S1**. Flow chart of the analysis pipeline**.** The RNA seq data from GEO was further filtered based on the clinical information and subjected to the upstream RNA-seq analysis pipeline, which includes quality control, trimming, alignment, quantification, and differential expression. The tools used for each of the above-mentioned steps are labeled inside the yellow box, besides the blue box. The DE genes were subjected to pathway enrichment using clusterProfiler. Additionally, the RNA-seq expression data was used to perform WGCNA analysis (green color box). The DE genes in the correlated modules were subjected to pathway enrichment and geneMANIA analysis to uncover potential PPI and TF networks. Besides the RNA-seq data, melanoma ChIP-seq data was used to identify SE associated genes. The overlap between SE associated genes and the correlated gene clusters were assessed and subjected to TF enrichment analysis (pink color box). DE: differentially expressed; TF: transcription factor; SE: super-enhancer.

**
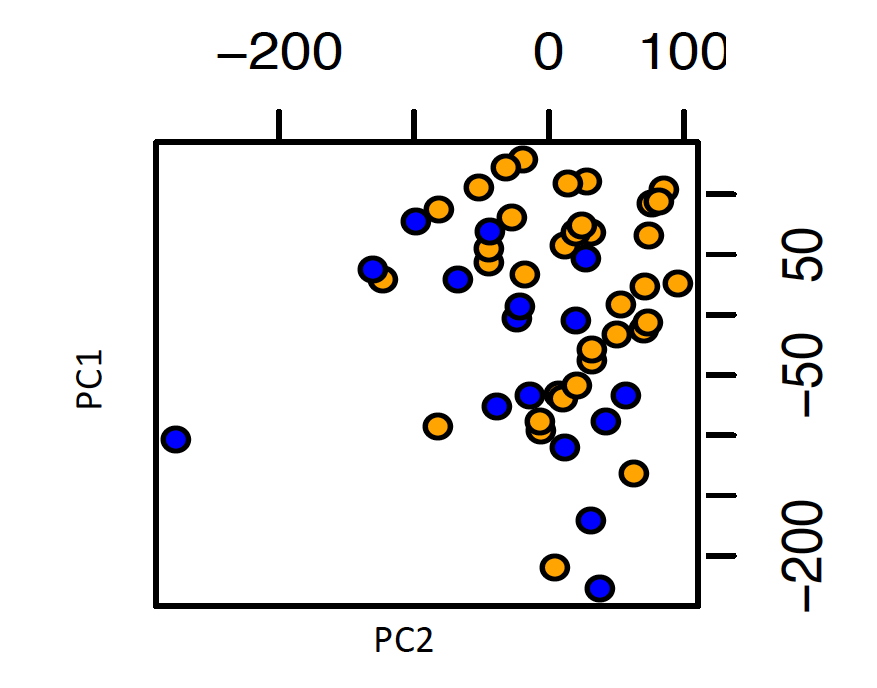
 Figure S2.** PCA plot of DE gene’s expression data from unfiltered invasive versus non-invasive RNA-seq analysis.

**
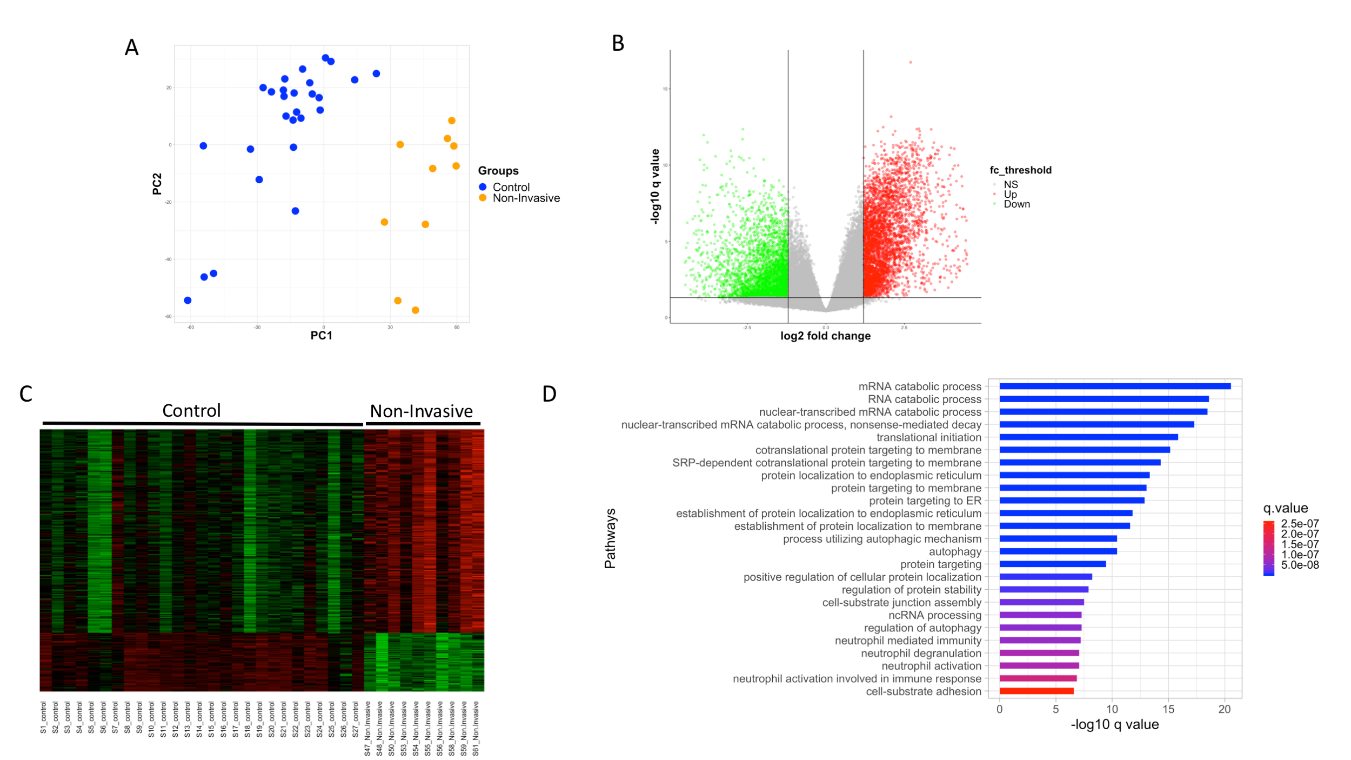
 Figure S3.** RNA-seq analysis of control versus stage 1 (non-invasive/early). (**A)**. PCA plot of DE gene’s expression data from control versus stage 1 (non-invassive) gene expression analysis. (**B)**. Volcano plot showing foldchange information of all genes from control versus stage 1 comparison. The statistically significant upregulated genes and downregulated genes are colored red and green, respectively. (**C)**. Heatmap of top 250 DE genes for the indicated groups labeled at the top of the plot. **D**. Top 25 pathways in which the DE genes from control vs. stage 1 comparison enriched for. PCA: principal component analysis; DE: differentially expressed.

**
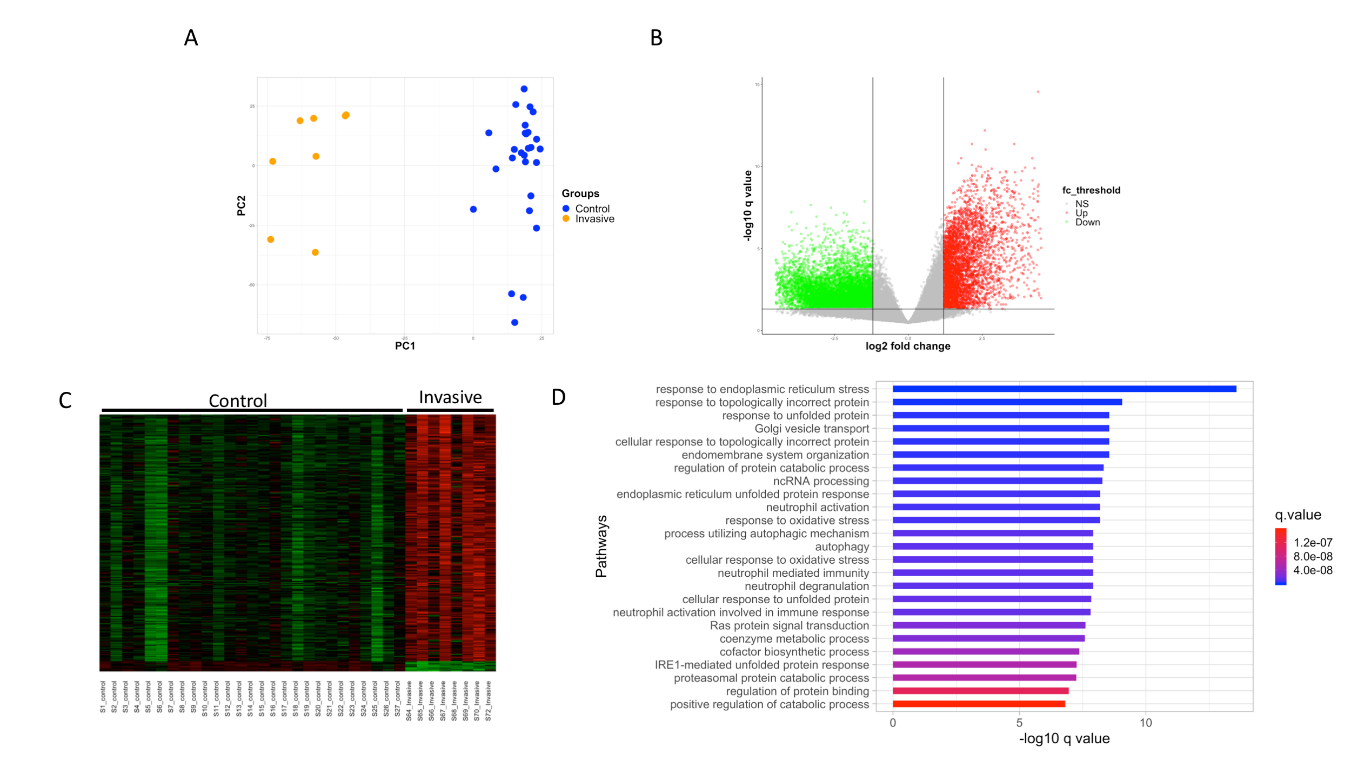
 Figure S4.** RNA-seq analysis of control versus stage 4 (invasive/late). **(A)**. PCA plot of DE gene’s expression data from control versus stage 4 gene expression analysis. (**B)**. Volcano plot showing foldchange information of all genes from control versus stage 4 comparison. The statistically significant upregulated genes and downregulated genes are colored red and green, respectively. (**C)**. Heatmap of top 250 DE genes for the indicated groups labeled at the top of the plot**. (D)**. Top 25 pathways in which the DE genes from control vs. stage 4 comparison enriched for. PCA: principal component analysis; DE: differentially expressed.

**
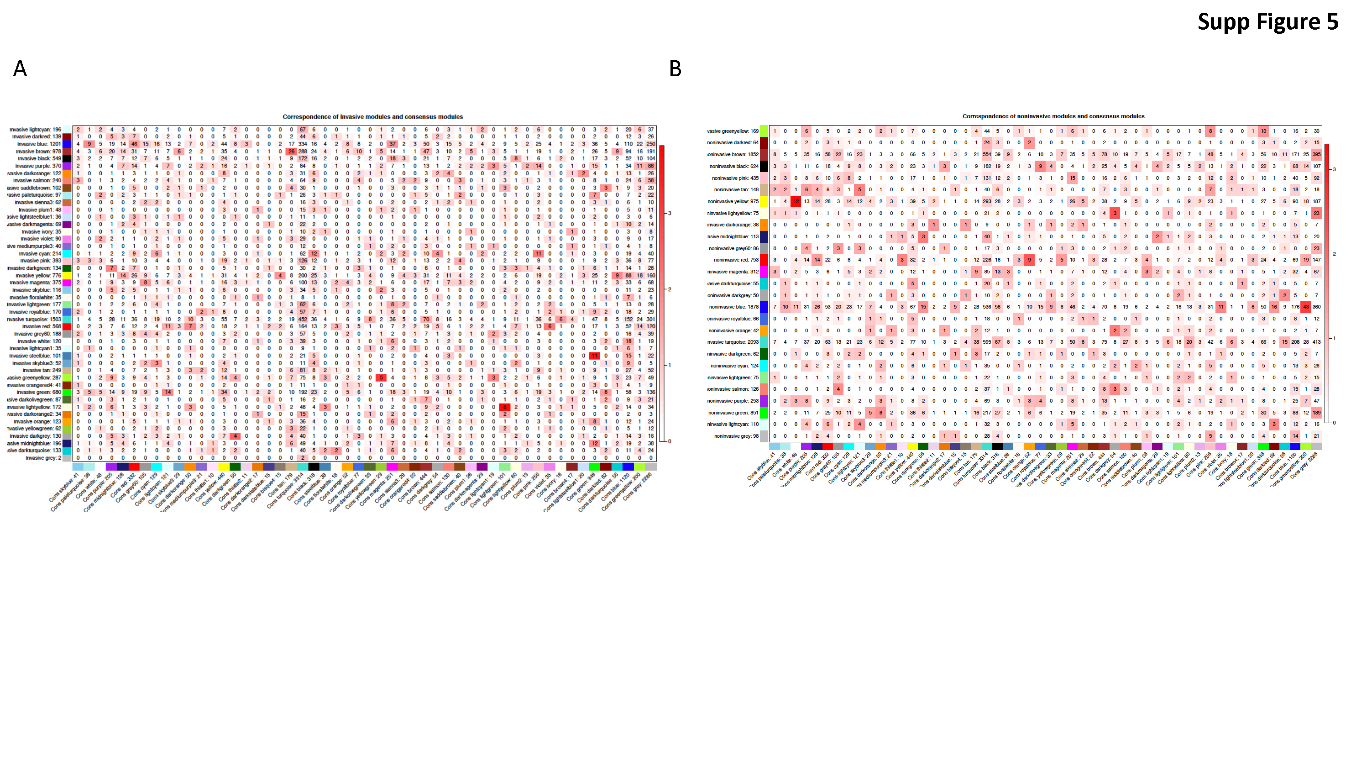
Figure S5.** Overlap of consensus co-expression modules with invasive and non-invasive RNA-seq modules**. (A)**. The x-axis represents co-expression modules from consensus WGCNA analysis. The y-axis represents co-expression modules from WGCNA analysis of the invasive (stage 4) RNA-seq data. The numbers in the plot represents number of common genes present for the indicated modules from x and y-axes. Significant overlaps are colored in shades of red, with darker shade of red indicating lower p values. (**B)**. The x-axis represents co-expression modules from consensus WGCNA analysis. The y-axis represents co-expression modules from WGCNA analysis of the non-invasive (stage 1) RNA-seq data. The numbers in the plot represents number of common genes present for the indicated modules from x and y-axes. Significant overlaps are colored in shades of red, with darker shade of red indicating lower p values.

**
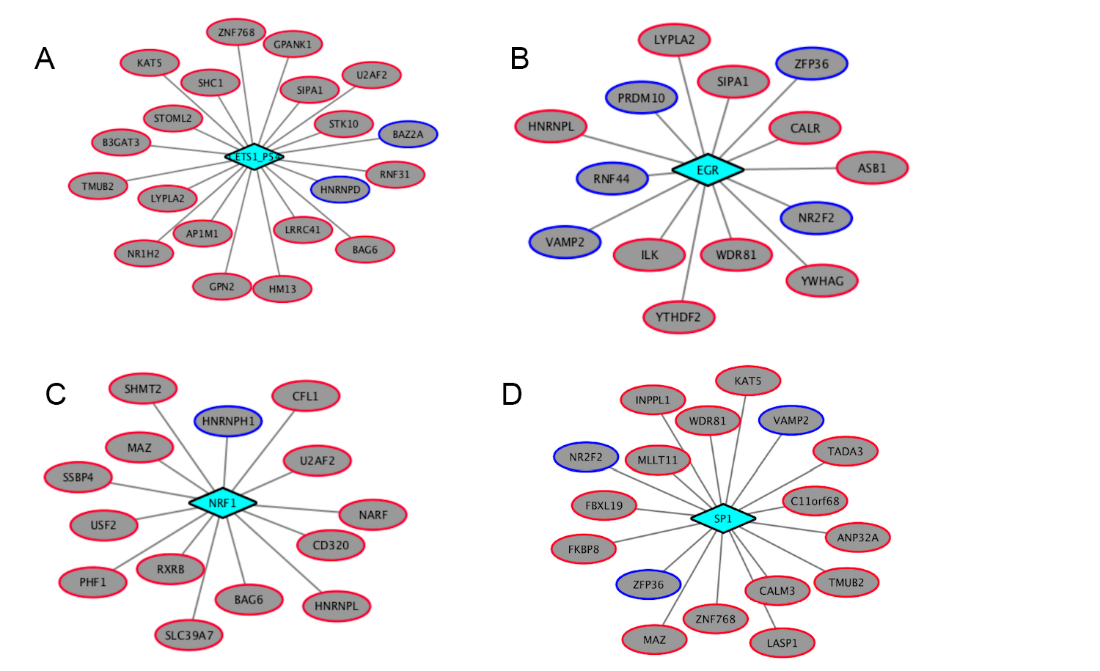
**

**Figure S6.** TF networks identified from genes in the Turquoise module that overlapped with SE **genes.** The diamond shaped node represents the TF. The spokes connect the TF with target genes represented as oval-shaped nodes. The red and blue color border of the target genes indicate that they are up and down regulated in stage 4 (late/invasive) melanoma, respectively. A. TF network for ETS1 transcription factor. B. TF network for NRF1 transcription factor. C. TF network for EGR transcription factor. D. TF network for SP1 transcription factor.
